# Phenotypic and Molecular Assessment of Drug Resistance Profile and Genetic Diversity of Waterborne *Escherichia coli*

**DOI:** 10.1007/s11270-016-2833-z

**Published:** 2016-04-19

**Authors:** Katarzyna Wolny-Koładka, Anna Lenart-Boroń

**Affiliations:** Department of Microbiology, University of Agriculture in Cracow, Mickiewicza Ave 24/28, 30-059 Cracow, Poland

**Keywords:** *Escherichia coli*, Water, Antibiotic resistance, Disk-diffusion method, ESBL, PCR

## Abstract

Bacterial *Escherichia coli* isolates were derived from waters of the Nowohucki Reservoir (Cracow, Poland) in summer and winter seasons. In total, 94 strains, identified as *E. coli*, were isolated from five sampling sites in the area of the reservoir. Based on the disk-diffusion tests, it was found that the tested isolates were predominantly resistant to ticarcillin and ampicillin. Numerous multi-drug resistant strains were detected, which however did not exhibit the ESBL phenotype. However, PCR approach allowed to detect ESBL-mechanism genes (CTX-M3, OXA, SHV, and TEM) in as much as 38 % of *E. coli* isolates. These results were coupled with significant molecular diversity of the *E. coli* strains revealed in BOXA1R-based rep-PCR technique.

## Introduction

The increasing microbial drug resistance is one of the most serious problems of modern medicine. This is why numerous steps are being taken in order to carefully examine the mechanisms of its formation and to assess the scale of the issue. Bacterial resistance can be natural (characteristic of particular species) or acquired (specific to particular microbial strain). Moreover, resistance to antibiotics is usually affected by many different mechanisms. Natural drug resistance often results from a lack of target sites for antibiotics in the cells of a given species or the presence of structures blocking its reach the target site. Though, in theory, natural resistance does not constitute a serious clinical problem. In contrary, induced drug resistance may constitute much more severe problem when we consider great ability of bacteria to acquire DNA from environment. With this ability, they can capture new genes or other DNA fragments that may induce rearrangements in their chromosomal DNA and thus change the expression of their own genes. This mechanism, namely the horizontal gene transfer, is the way how bacteria can acquire new traits from even phylogenetically distinct organisms. Obviously, in that case, we are dealing with acquired resistance that leads to the occurrence of bacterial strains that are resistant to antimicrobial treatment. Additionally, strong selection pressure, resulting from the excess use of antibiotics, causes the selection of strains being resistant to multiple pharmaceuticals. Multi-drug resistant strains are characterized by the presence of various resistance genes, which make them insensitive to many different groups of drugs (Livermore and Williams 2000; Rozenberg-Arska and Visser 2004; Schmitz and Fluit 2004).

Among numerous existing antibiotic resistance mechanisms in the family of *Enterobacteriaceae*, which comprises also *Escherichia coli*, particular attention should be paid to the production of extended spectrum β-lactamases (ESBL). Strains, in which the ESBL resistance mechanism was found, are capable of hydrolysis of all penicillins, cephalosporins (except cephamycin), and monobactams (Bush et al. 1995). The ability to produce extended spectrum β-lactamases may be acquired as genes responsible for the formation of ESBL mechanism are located on plasmids. Additionally, genes encoding ESBLs are rapidly spread, also among strains that do not belong to the same species, which is due to their location on conjugation plasmids (e.g., IncFII and IncI) (Hopkins et al. 2006; Novais et al. 2007; Coque et al. 2008; Marcade et al. 2009). This is why strains having acquired ESBL, which was first identified in 1983, are now one of the most serious clinical problems worldwide (Knothe et al. 1983; Kliebe et al. 1985). The ESBL mechanism is most commonly found among hospital strains from *Enterobacteriaceae* family. However, invasive *E. coli* strains capable of extended spectrum β-lactamase production have been also identified from other environments in recent years (Paterson and Bonomo 2005; Canton et al. 2008).

It is a well known fact that *E. coli* occurs mainly in the gastrointestinal tract of humans and animals. Therefore, it is also present in natural environment especially in water, soil, and on plants. The spread of *E. coli* in the environment is also affected by discharge of municipal sewage into surface water and soil (Watkinson et al. 2007). *E. coli* is also widely recognized indicator of microbiological purity of water. However, increasing attention is paid to its role in spreading antibiotic resistance in water environment (Baquero et al. 2008). This is particularly important, because the detection of antibiotic resistant *E. coli* may be indicative of the presence of allochtonic pollutants in the aqueous environment, which is especially useful in monitoring studies (Bartoszewicz et al. 2014).

The object of this study was the Nowohucki Reservoir, which as artificial water reservoir was put into service in 1957 and became a leisure site for the residents of the old part of Nowa Huta (district of Cracow, Poland). The reservoir was built within a 2-km green protection zone, separating the steelworks factory (former Tadeusz Sendzimir Steelworks, currently ArcelorMittal Poland JSC) from the housing estates of Nowa Huta. In total, this area covers approx. 17 ha, with the basin area of over 7 ha (Dzieszyński and Franczyk 2006). In the 1950s and 1960s of the twentieth century, the Nowohucki Reservoir was a place of rest and recreation for the residents of this district. Later on, this area has been neglected and currently the reservoir has four basic functions: it is a fishery managed by the Fishing Circle, place of breeding and growth of aquatic birds, protected amphibians and reptiles, as well as an unauthorized bathing area and the reservoir which supplies the nearby allotment gardens. Waters of this reservoir are not subject to sanitary and hygienic assessments conducted by the State Sanitary Inspectorate. It should be noted that, despite clear bathing prohibition, hundreds of people swim and rest by this reservoir during the holiday season each year.

The aim of this study was to isolate and to confirm the systematic position of waterborne *E. coli* strains from the Nowohucki Reservoir. In addition, the antimicrobial resistance profile of strains was determined and molecular marker-based analyzes were applied to diversify the *E. coli* isolates obtained in two periods (summer and winter) at five sites situated within the Nowohucki Reservoir. Information obtained in this study will help to determine whether water of the examined reservoir contains multidrug resistant strains of *E. coli* that can pose epidemiological threat. Moreover, the use of both phenotypic and molecular methods will enable the comparison of their sensitivity and reproducibility.

## Material and Methods

### Collection of Water Samples

**Fig. 1:**
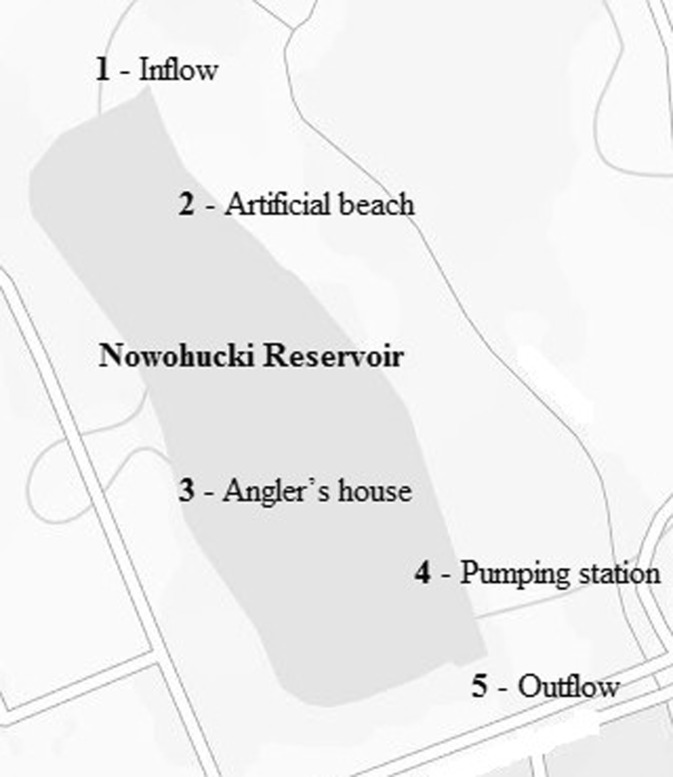
Location of water sampling sites (www.mapy.geoportal.gov.pl/imap/)

The water samples were collected from five points situated within the Nowohucki Reservoir, located in Nowa Huta—the largest district of Cracow (Poland). The samples were taken into sterile containers, twice per year on the following dates: January 15th 2015 (winter) and August 8th 2015 (summer). The collection points are presented in Fig. 1: site 1—water inflow; sites 2, 3, and 4—characteristic sites along the reservoirs banks; and 5—outflow. Water and air temperature were measured onsite during sampling using an electronic thermometer (Biowin). The isolation of *E. coli* was conducted by membrane filtration method on ENDO agar (BTL, Poland). The cultures were incubated at 44 °C for 48 h to isolate thermotolerant (fecal) *E. coli* (purple colonies with metallic sheen).

### Isolation and Identification of *E. coli*

In order to confirm the species identification of the collected bacterial isolates, the purple colonies with metallic sheen were transferred from ENDO agar onto selective, chromogenic TBX agar (Tryptone Bile Agar with X-Glucuronide–BTL, Poland) and incubated at 44 °C for 24 h, followed by Gram staining of microscopic preparations. Colonies, microscopically identified as gram-negative rods and forming green-blue colonies on TBX agar, were propagated and used in further analyses.

### Antimicrobial Resistance, Disk-Diffusion Method

The susceptibility of waterborne *E. coli* strains to antibiotics was determined with disk-diffusion tests on Mueller-Hinton agar (BTL, Poland). The evaluation was conducted according to the National Reference Centre for Antimicrobial Susceptibility Testing (KORLD), given in the “Recommendations for the selection of tests for susceptibility of bacteria to antibiotics and chemotherapeutics, 2009. Determination of the susceptibility of Gram-negative rods” (Gniadkowski et al. 2009). According to the guideline, the analysis was conducted using 20 antibiotics included in both basic and extended antibiogram (Table 1).

**Table 1:** Applied antibiotic disks (Oxoid)

No.	Antibiotic	Abbr.	Concentration (μg)	Class
1.	Amikacin	AK	30	Aminoglycosides
2.	Amoxicillin/clavulanic acid^a^	AMC	30	3rd generation penicillins
3.	Ampicillin	AMP	10	Aminoglycosides
4.	Aztreonam	ATM	30	Monobactams
5.	Cefamandole	MA	30	2nd generation cephalosporins
6.	Cefepime	FEP	30	4th generation cephalosporins
7.	Cefotaxime^a^	CTX	30	3rd generation cephalosporins
8.	Cefoxitin	FOX	30	3rd generation cephalosporins
9.	Ceftazidime^a^	CAZ	30	3rd generation cephalosporins
10.	Cefalotin	KF	30	3rd generation cephalosporins
11.	Cefazolin	KZ	30	1st generation cephalosporins
12.	Ciprofloxacin	CIP	5	Chinolons
13.	Gentamicin	CN	10	Aminoglycosides
14.	Netilmicin	NET	30	Aminoglycosides
15.	Piperacillin	PRL	100	4th generation penicillins
16.	Piperacillin/tazobactam	TZP	110	4th generation penicillins
17.	Tetracycline	TE	30	Tetracyclines
18.	Ticarcyillin	TIC	75	4th generation penicillins
19.	Tobramycin	TOB	10	Aminoglycosides
20.	Trimethoprim/sulfamethoxazole	SXT	25	Sulfonamides

^a^Antibiotics used in the detection of ESBL mechanism

*E. coli* reference strain ATCC 25922 was used as control—this strain is used for quality control in the susceptibility testing and for the quality control in the ESBL mechanism assessment as a negative control. *E. coli* ATCC 35218 was also used in order to test the quality of antibigram disks containing β-lactam antibiotics in combination with β-lactamase inhibitors (clavulanic acid, sulbactam, and tazobactam).

### DNA Extraction and PCR Assays

DNA of *E. coli* isolates was extracted using Genomic Mini kit (A&A Biotechnology, Poland) according to the manufacturer’s instructions and was used as a template for all PCRs.

The PCR tests were applied for the detection of ESBL genes, namely blaCTXM3 (Costa et al. 2006), blaCTXM9 (Simarro et al. 2000), blaOXA, blaSHV, and blaTEM (Sáenz et al. 2004). PCR reactions were carried out in a 25 μl volume containing 50 ng of DNA template, 12.5 pM of each primer, 2.5 mM of dNTP, 1× PCR buffer, and 1 U of DreamTaq DNA polymerase (ThermoScientific, USA). The following temperature profile was used for the reactions: initial denaturation at 95 °C for 5 min, followed by 35 cycles of 94 °C for 45 s, annealing for 45 s at different temperatures, then extension at 72 °C for 1 min with final extension at 72 °C for 10 min and then stored at 4 °C. PCR amplifications were performed in T100^TM^ Thermal Cycler (Bio-Rad, USA). The PCR products were electrophoresed for 60 min in 1× TBE Simply Safe (EurX, Poland)-stained (0.5 mg/ml), 1 % agarose gel, visualized in UV light and documented by the GelDoc system (BioRad, USA).

Molecular differentiation of bacterial strains was conducted with the rep-PCR technique using BOXA1R primer (Versalovic et al. 1994). All reactions were performed in two replicates. PCR reaction was performed in a total volume of 25 μL containing approximately 20 ng of DNA template, 12.5 pM of the primer, 2.5 mM of dNTP, 1× PCR buffer, and 1 U of DreamTaq DNA polymerase (ThermoScientific, USA). The PCR amplification was performed in T100^TM^ Thermal Cycler (Bio-Rad, USA) using the following temperature profile: initial denaturation at 94 °C for 5 min, followed by 25 touchdown cycles of denaturation at 94 °C for 30 s, annealing starting from 67.5 °C with temperature decreasing by 0.5 °C in each cycle until 55 °C for 30 s and elongation at 72 °C for 1 min and then 20 cycles of denaturation at 94 °C for 30 s, annealing at 55 °C for 30 s and elongation at 72 °C for 1 min, and final elongation for 10 min. The PCR products were electrophoresed for 120 min in 1.5 % agarose gel, stained with Simply Safe (EurX, Poland) up to 0.5 mg/ml in 1× TBE buffer. After the electrophoresis, the gel was analysed with UV light and GelDoc (Applied Biosystems, USA). Rep-PCR results were scored on agarose gels for two independent replicates. The bands present on both gels were scored and encoded in the presence-absence binary matrix.

### Numerical Analysis

*χ*^2^ test was performed in order to verify the significance of differences in the antimicrobial resistance between *E. coli* strains isolated in two dates (summer and winter). Statistical analysis was performed using the Social Science Statistics calculator (http://www.socscistatistics.com/tests/chisquare/Default.aspx).

FaBox (Villesen 2007) was used to verify the presence of clonal strains in the Rep-PCR analysis by searching for individual haplotypes. Strains carrying the same Rep-PCR haplotype were considered clonal in our analysis. The intra- and inter-population variation was assessed using AMOVA analysis carried out using Arlequin 3.1.1 (Excoffier and Lischer 2010) with Rep-PCR data coded as standard datatype.

The genetic distances between strains were calculated based on the binary matrix of amplified fragments, and Unweighted Pair Group Method with Arithmetic Mean (UPGMA) dendrograms was constructed using SplitsTree 4 (Huson and Bryant 2006).

## Results

The pool of 94 *E. coli* strains isolated from water of the Nowohucki Reservoir included 44 strains isolated in winter and 50 strains in summer. During sampling, water and air temperature was measured onsite and the results are given in Table 2.

**Table 2:** Water and air temperature measured at the sampling sites

No.	Sampling site	Winter—January 15th 2015		Summer—August 10th 2015	
		Air temp. (°C)	Water temp. (°C)	Air temp. (°C)	Water temp. (°C)
1.	Inflow	1.6	4.1	28.1	19.1
2.	Artificial beach	0.8	1.8	25.7	26.8
3.	Angler’s house	1.3	3.8	26.1	27.9
4.	Pumping station	0.6	1.5	26.3	26.3
5.	Outflow	0.7	3.9	23.2	18.5

Disk-diffusion tests allowed to determine the resistance of bacterial isolates to antimicrobials and the results are shown in Table 3. The resistance to ticarcillin (53 resistant strains) and ampicillin (50 resistant strains) was most frequently found. High percentage of resistant strains was also found in the case of cephalothin, cefazolin, and amoxicillin with clavulanic acid. No resistance to amikacin and piperacillin-tazobactam was observed. Highly susceptible strains to netilmicin, cefepime, cefotaxime, trimethoprim-sulfamethoxazole, piperacillin, and ceftazidime were detected. The ESBL-type resistance mechanism was not observed.

**Table 3:** The share of *E. coli* isolates—sensitive, intermediate sensitive, and resistant to the examined antibiotics

No.	Antibiotic	(S) Sensitive	(I) Intermediate	(R) Resistant
1.	Amikacin	91	3	0
2.	Amoxicillin/clavulanic acid	63	0	31
3.	Ampicillin	44	0	50
4.	Aztreonam	71	5	18
5.	Cefamandole	72	1	21
6.	Cefepime	86	6	2
7.	Cefotaxime	84	3	7
8.	Cefoxitin	66	0	28
9.	Ceftazidime	81	7	6
10.	Cefalotin	42	17	35
11.	Cefazolin	41	18	35
12.	Ciprofloxacin	71	3	20
13.	Gentamicin	61	28	5
14.	Netilmicin	88	2	4
15.	Piperacillin	81	0	13
16.	Piperacillin/tazobactam	93	1	0
17.	Tetracycline	61	11	22
18.	Ticarcillin	41	0	53
19.	Tobramycin	53	34	7
20.	Trimethoprim/sulfamethoxazole	82	4	8

**Fig. 2:**
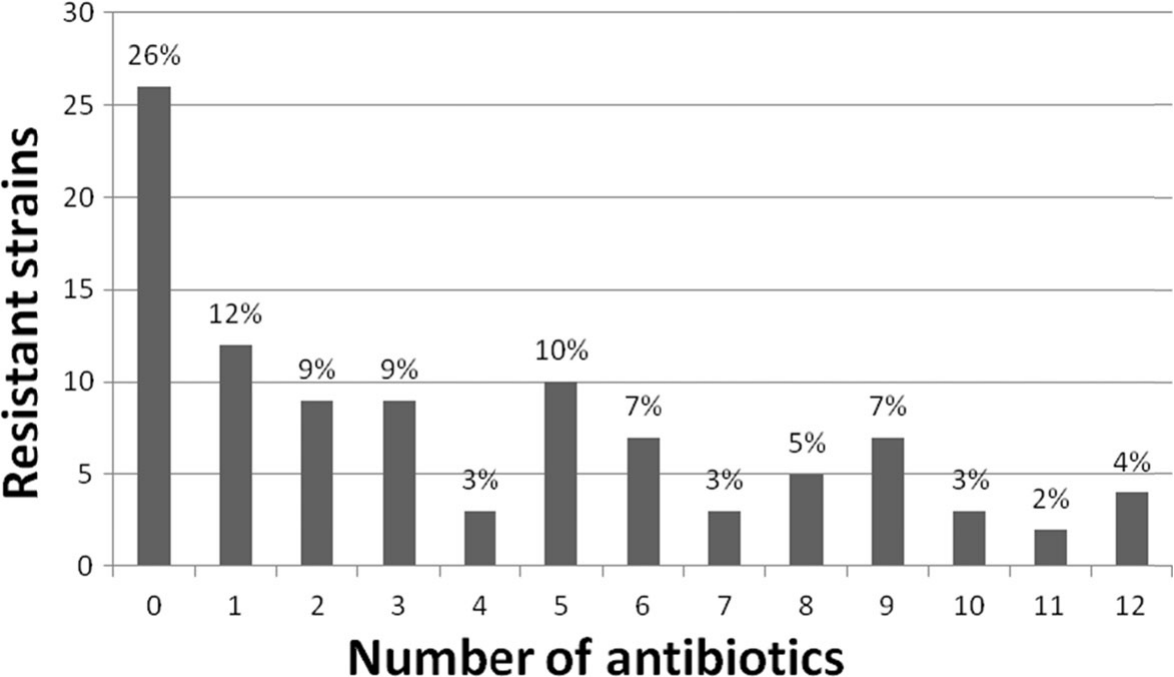
The proportion of multi-drug resistant *E. coli* strains

Among the collected *E. coli* strains, 26 % were sensitive to all antibiotics tested. However, the remaining strains (74 %) were resistant to one or more antibiotics and 4 % of the isolates were resistant to even 12 antibiotics at the same time (Fig. 2).

**Fig. 3:**
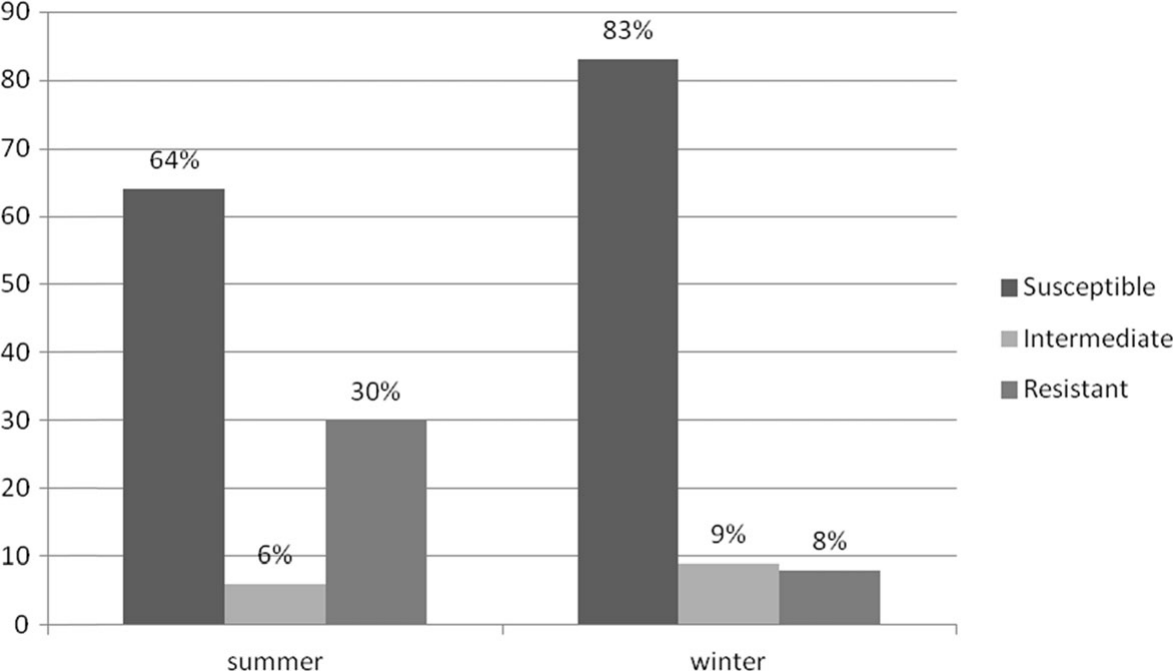
Changes in the waterborne *E. coli* antimicrobial resistance profile according to the period of strain isolation

The analysis of antimicrobial resistance profile of waterborne *E. coli* isolated from the Nowohucki Reservoir indicated that the strains collected during the summer are characterized by much higher resistance to the tested drugs (Fig. 3).

**Fig. 4:**
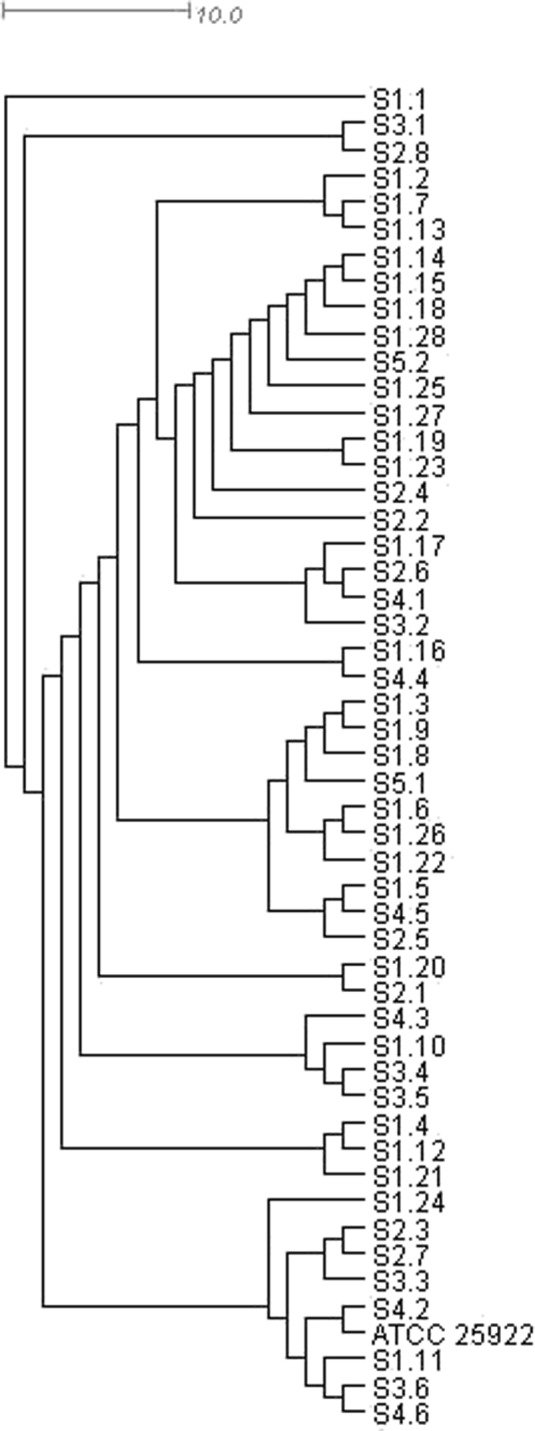
UPGMA dendrogram of waterborne *E. coli* strains collected in the summer from 5 points located within the area of the Nowohucki Reservoir

**Fig. 5:**
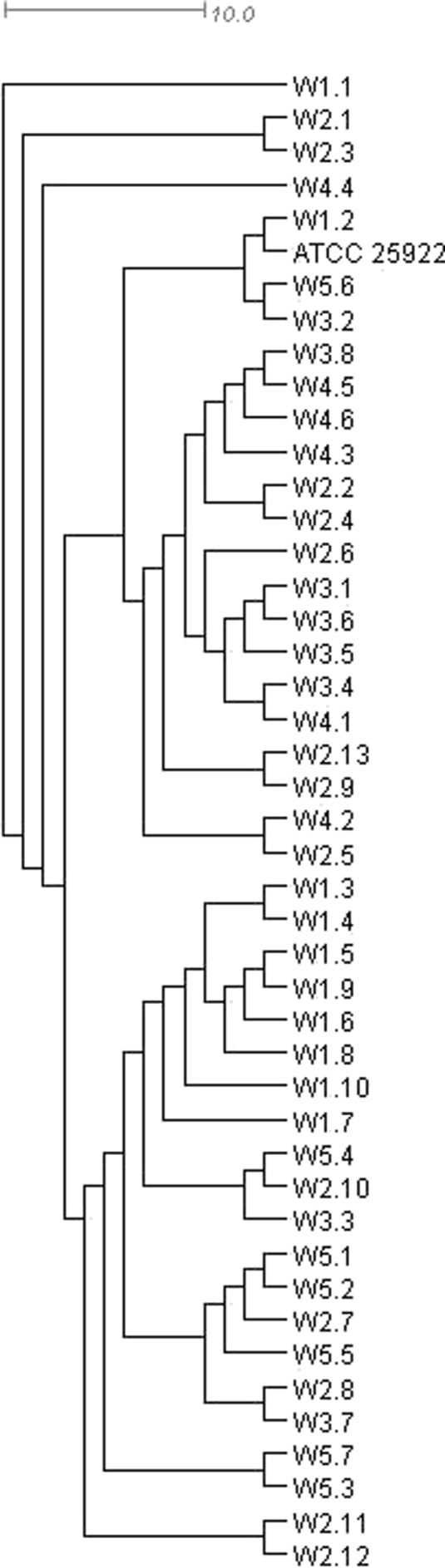
UPGMA dendrogram of waterborne *E. coli* strains collected in the winter from 5 points located within the area of the Nowohucki Reservoir

Dendrograms (Figs. 4 and 5), which showed the results of analyses aiming at determining most closely related isolates, as well as verification of their potential relationship with the reference strain, were constructed based on the rep-PCR analyses (BOX-PCR). The figures show significant diversity between *E. coli* isolates. Based on these dendrograms, it can be concluded that the examined isolates are characterized by large genetic diversity and they cannot be easily grouped into clusters. Some clusters are composed of isolates derived from individual sampling sites, while others comprise isolates from various sites. Also, the position of a reference strain ATCC 25922 does not allow to demonstrate significant differences between the clusters obtained. The analysis of the presented dendrograms allows to conclude that the *E. coli* strains, even though originate from a relatively isolated environment, such as the tested reservoir, are very diverse. The overall genetic variation between and within groups (summer and winter) according to AMOVA principles was presented in Table 4.

**Table 4:** The results of AMOVA analysis for *E. coli* isolates in two groups (summer and winter) with five populations (sampling sites) each

Source of variation	Sum of squares	Variation components	Percentage of variation
Among groups	14.119	0.13679	4.00
Among populations within groups	46.513	0.34889	10.20
Within populations	246.528	2.93486	85.80
Total	307.160	3.42054	100.00
Fixation index—Fst	0.14199		

The analysis of the occurrence of specific PCR products in the electrophoretic image indicates that the genes responsible for the emergence of the ESBL type of resistance occur in 36 out of 94 strains (38 %). Furthermore, larger numbers of the examined genes were found in bacteria isolated in summer (*n* = 22, 61 %), than those isolated in winter (*n* = 14, 39 %).

**Fig. 6:**
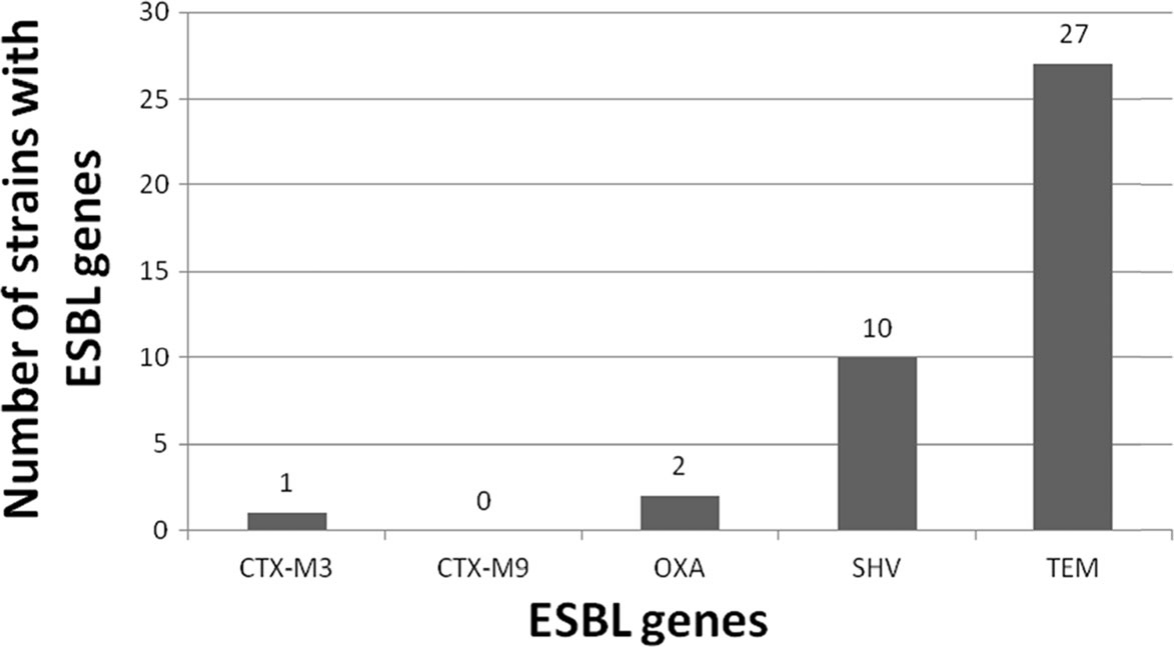
Prevalence of antibiotic resistance genes responsible for the emergence of ESBL mechanism in *E. coli*

Figure 6 shows the incidence of genes responsible for the production of extended spectrum β-lactamases (ESBL). The *blaTEM* gene was most frequently found (occurred in 27 isolates), while *blaCTX-M9* was not detected in any of the analyzed strains. In most cases, the analyzed genes occurred separately in the *E. coli* strains, only in two isolates we found a co-occurrence of two genes, i.e., *blaOXA* with *blaTEM* and in another two—*blaSHV* with *blaTEM*. Moreover, the presence of blaTEM only was found in bacteria isolated in the winter, while the remaining genes were present in *E. coli* strains isolated in the summer.

Statistical analysis was performed to assess the significance of differences in the resistance to individual antibiotics between *E. coli* strains isolated in two study periods (summer and winter). The *χ*^2^ test confirmed that the differences in the prevalence of isolates resistant to 10 out of 20 of the tested antibiotics are statistically significant (*p* < 0.05). The value of *χ*^2^ statistics was as follows: amoxicillin with clavulanic acid (7.4087), ampicillin (22.3189), aztreonam (14.1469), cefamandole (18.7699), cefoxitin (12.4962), cephalothin (19.651), cefazolin (20.5979), tetracycline (7.775), ticarcillin (24.2265), and trimethoprim-sulfamethoxazole (8.0332).

## Discussion

Currently in Poland as much as 10 % of untreated municipal sewage and approximately 30 % of insufficiently treated sewage is discharged into surface water. Therefore, the constant presence of bacterial sewage contamination indicators, such as *E. coli* in surface water, is not surprising. The main aim of microbiological analyzes of surface water quality is to assess the number of *E. coli*, which is a widely recognized indicator of fecal contamination of water. Nevertheless, the problem of resistance of environmental *E. coli* strains to antibiotics commonly used in medicine becomes also increasingly important (Bartoszewicz et al. 2014).

Based on the conducted disk-diffusion tests, it was found that the *E. coli* isolates were characterized by the greatest resistance to ticarcillin and ampicillin, 56 and 53 % of resistant strains, respectively. Pignato et al. (2009), Reinthaler et al. (2003), and Patoli et al. (2010) in their studies found that the antibiotic to which waterborne *E. coli* strains were most frequently resistant was ampicillin (88.89, 22.71, and 18 % resistant strains). Sana et al. (2012), while studying the sensitivity of clinical *E. coli* strains to ticarcillin and ampicillin, observed 100 % resistance to these two antibiotics. On the other hand, we observed no strains resistant to amikacin, similarly as in the studies by Patoli et al. (2010). In our study, similarly as in Martinez et al. (2012), there was a large number of strains susceptible to ceftazidime and cefotaxime, whereas Patoli et al. (2010) consider these as antibiotics against which *E. coli* shows high resistance. Moreover, based on the obtained results also 37 % of cephalothin resistance was found, which is also consistent with the results of Reinthaler et al. (2003), who observed the 35 % of resistance to this antibiotic. Similar results as Reinthaler et al. (2003) were also obtained with respect to the susceptibility to piperacillin and trimethoprim/sulfamethoxazole. In our study, there were 86 and 87 % strains susceptible, respectively, while in the study by Reinthaler et al. (2003), 88 and 87 %.

Phenotypic analyses, although did not allow for the detection of ESBL strains, showed the presence of multi-drug resistant isolates. Multi-drug resistant, non-ESBL-producing strains were resistant to even 12 antimicrobials at the same time, including cephalosporins, whose administration should be limited only to justified cases, due to their proven role in the selection of multi-drug resistant isolates. Such high antimicrobial resistance is rarely detected. It is another argument for the need to monitor the susceptibility of *E. coli* derived from the hospital environment (Kot et al. 2010). In our study, the resistance to two or more antibiotics was observed in 62 % strains. Sahm et al. (2001) found that the strains resistant to 4 (1.4 %) or 5 (0.2 %) antimicrobials are much less frequent than those resistant to 1 (20 %) or 2 (17 %). A similar relationship could be found in research by Patoli et al. (2010), where the resistance to two antibiotics was observed in 33.34 % of isolates, while the resistance to five antibiotics was only in 3.70 %, whereas it should be noted that as much as 11.11 % of *E. coli* strains were resistant to six antibiotics.

Among 94 isolates collected in these analyses, 36 had *blaCTX-M3*, *blaOXA*, *blaSHV*, and *blaTEM* genes, responsible for the emergence of ESBL type of resistance. Martinez et al. (2012), when analyzing the prevalence of ESBL resistance phenotype in *E. coli* responsible for urinary tract infections, found this type of resistance in 11.7 % of isolates. On the other hand, the presence of genes involved in the formation of ESBL resistance mechanism (*blaTEM*, *blaSHV*, *blaCTX*, *blaFER*, and *blaCMY*) was found at the following level: TEM 6.9 %, CTX 6.9 %, and CMY 8.8 %, while the remaining genes were not detected. In the study by Zaniani et al. (2012), ESBL-type *E. coli* strains were isolated from urine and blood of hospitalized patients comprised as much as 43.9 %, while the genes *blaSHV* and *blaTEM* were detected in 14.4 and 20.6 % isolates, respectively. Sana et al. (2012), while examining the presence of ESBL genes in 73 clinical, ESBL-producing *E. coli* strains, found the following prevalence: CTX-M 98.6 %, OXA 45.2 %, TEM 21.9 %, and SHV 4.1 %.

The observations collected in our research indicated that the strains isolated in summer are characterized by much higher resistance to the tested antibiotics. This is probably due to the fact that despite the prohibition of bathing, hundreds of people as well as dogs swim in this reservoir at this time of year. On the other hand, there was no relationship between the water sampling site and the antimicrobial resistance profile of *E. coli* isolated from the particular site. There is excessive mixing of water in the reservoir itself, as well as at its inflow and outflow, which most probably has large impact on the movement of bacteria within the reservoir. Moreover, the measurement sites are not very distant from each other (several up to a few hundred meters away) which make it difficult to find a specific pattern within the drug resistance variation between the *E. coli* strains isolated in individual sites.

These observations are supported by the results obtained from the analysis of molecular diversity between bacterial isolates. According to AMOVA results, the variation within populations covered 85.80 % of overall variation. It is relatively high proportion as compared with the variation between populations (sites) and even more when variation among groups (summer vs. winter) is concerned, which may suggest that the bacterial strains originate from various sources of contamination, both in different seasons of the year and in individual sampling sites (populations within groups). This indicates that statistically significant difference in the antimicrobial resistance between summer and winter isolates is not accompanied by clear genetic diversity pattern. Thus, higher resistance of summer strains does not result from their common origin, but other factors are most probably involved. These factors were not identified in this research, but their overall effect results in higher selection pressure toward drug resistance in summer than in winter.

Currently, the production of extended spectrum β-lactamases (ESBL) is one of the most important mechanisms of drug resistance from a clinical and epidemiological point of view. Despite constantly emerging new resistance mechanisms, it is the ESBL mechanism that remains still the most common cause of *E. coli* resistance to penicillins, most cephalosporins and monobactams (Bush et al. 1995; Bradford 2001; Canton et al. 2008; Cornaglia et al. 2008; Empel et al. 2008). A few years back, the ESBL strains were considered typical nosocomial pathogens. However, recent reports show that the ESBL strains are identified also outside this environment, as etiological factors of community-acquired infections (particularly urinary tract infections) and they are found in people being their carriers only, in animals and food products (Pitout et al. 2005; Canton et al. 2008; Carattoli 2008). Therefore, even though no ESBL strains were found in the disk-diffusion test, it is reasonable to continue identifying this mechanism based on the antibiogram in all *Enterobacteriaceae* isolates derived from different environments. This is very important from an epidemiological point of view and will facilitate monitoring and control of potential infections (Gniadkowski et al. 2009). The results of our study indicate that it needs to be remembered that despite the lack of phenotypically proven ESBL mechanism, the genes responsible for its emergence were detected in the examined *E. coli* isolates. It is a well known fact that despite the presence of genes affecting the production of certain enzymes, i.e., extended spectrum β-lactamases, the disk-diffusion test does not indicate the occurrence of any specific ESBL mechanism (Kobayashi et al. 1995; McDonald et al. 1995; York et al. 1996; Idzik et al. 2000; Wolny-Koładka et al. 2014). The results obtained in this study confirm that there is a low correlation between the results of phenotypic and genetic analyses. This may be due to the fact that antimicrobial resistance is encoded by multiple genes, or that the genes—although present in *E. coli* genotype—were not expressed, as shown in the drug resistance analysis.

## Conclusions

The performed microbiological analyses resulted in isolation and identification of 94 waterborne *E. coli* strains inhabiting the Nowohucki Reservoir. The antimicrobial resistance profile of the collected isolates was determined using the disk-diffusion method and by using the PCR technique. On this basis, it was concluded that the bacterial isolates were most frequently resistant to ticarcillin and ampicillin. The presence of numerous multidrug resistant strains was detected among the collected bacteria, which were resistant to as many as 12 tested antimicrobials. The applied phenotypic method aimed to determine the bacterial susceptibility/resistance to antimicrobials did not detect the presence of ESBL mechanism. In contrast, the use of PCR technique with specific primers allowed to detect the presence of genes (CTX-M3, OXA, SHV, and TEM) responsible for the occurrence of this type of resistance in *E. coli*. Therefore, it can be concluded that detection of the ESBL mechanism using phenotypic methods may give false negative results. Having regard to the presented results of studies, as well as the fact that in summer the Nowohucki Reservoir is used for swimming by the local residents, it must be recognized that it would be reasonable to include waters of this reservoir into constant monitoring programme of sanitary authorities.
